# Emergent Management of Severe Hypothermia, Acidemia, and Coagulopathy in Operative Penetrating Ballistic Cranial Trauma

**DOI:** 10.7759/cureus.55630

**Published:** 2024-03-06

**Authors:** Nicholas Dietz, Meghan Blank, William Asaka, Brent G Oxford, Dale Ding, Emily Sieg, Heidi M Koenig

**Affiliations:** 1 Department of Neurosurgery, University of Louisville Hospital, Louisville, USA; 2 Department of Anesthesiology, University of Louisville Hospital, Louisville, USA

**Keywords:** decompressive craniectomy, neurocritical care, traumatic brain injury, coagulopathy, acidosis, hypothermia

## Abstract

Hypothermia in a trauma patient has been associated with increased morbidity and mortality and is more frequently seen in those sustaining traumatic brain injuries (TBIs). Acidosis is an important consequence of hypothermia that leads to derangements across the spectrum of the coagulation cascade. Here, we present a case of a 31-year-old male presented after suffering a right parietal penetrating ballistic injury with an associated subdural hematoma and 7 mm midline shift requiring decompressive craniectomy and external ventricular drain (EVD) placement in the setting of severe hypothermia (28°C) and acidosis (pH 7.12). With aggressive rewarming intraoperatively, the use of full-body forced-air warming, warmed IV fluids, and increasing the ambient room temperature, the patient’s acidosis and hypothermia improved to pH 7.20 and 34°C. Despite these aggressive attempts to rewarm the patient, he developed coagulopathy in the setting of concurrent hypothermia and acidosis. This case highlights the importance of prompt reversal of hypothermia due to its potentially fatal effects, particularly in the setting of severe TBIs. We discuss the critical aspects of surgical management of the injury and anesthetic management of hypothermia, acidosis, and coagulopathy perioperatively.

## Introduction

Hypothermia in a trauma patient has been associated with increased morbidity and mortality and is more frequently seen in those sustaining traumatic brain injuries (TBIs) [1-3]. Observed in as many as two-thirds of major trauma patients, hypothermia is defined as a core body temperature less than 36°C and is associated with a wide range of negative effects in the trauma patient, including coagulopathy, acidosis, cardiac arrhythmias and ischemia, increased peripheral vascular resistance, decreased drug metabolism, impaired wound healing, and a cascade of altered metabolic conditions [4,5]. To further classify, hypothermia may be mild (36-34°C), moderate (34-32°C), or severe (below 32°C) [1]. When the core body temperature measured at the tympanic membrane, nasopharynx, esophagus, and bladder decreases to 32°C, a host of cardiopulmonary abnormalities may ensue, including cardiac arrhythmias and cardiac ischemia [6]. By the time temperatures reach 28°C and below, heat production mechanisms begin to fail, and at 18-20°C, most patients succumb to asystole.

Risk factors for hypothermia in the critical trauma patient include an injury severity score greater than 15 (isolated critical head injury= ~26), environmental exposure, and hypovolemia from blood loss [4,7]. The present case demonstrated high risk from meeting all three of the above criteria. TBIs may disrupt physiologic thermoregulatory mechanisms that can exacerbate the deleterious effects of prolonged hypothermia and result in poor outcomes [5]. Normally, the hypothalamus maintains core temperature within a very narrow range, but this central thermoregulation by hypothalamic reflexes is inhibited by some anesthetics [8].

Acidosis is one consequence of hypothermia that includes derangements across the spectrum of the coagulation cascade and increases overall mortality and perioperative risk [1,3,9]. Specifically, fibrinogen and many coagulation factors are decreased, and this results in generalized hypocoagulability [10]. Treatment of this in isolation by giving sodium bicarbonate can be devastating for the patient without concomitant rewarming. Patients in the intensive care unit (ICU) have an increased risk of early and late death [3], in the setting of the lethal triad comprised of hypothermia, acidosis, and coagulopathy [5,11].

We present a case of traumatic brain injury secondary to penetrating ballistic cranial injury requiring emergent surgery in the setting of severe hypothermia. Intraoperative optimization requires efficient and comprehensive management of hypothermia, correction of potential coagulopathy, and practical considerations regarding further heat loss and active rewarming.

## Case presentation

**Figure 1:**
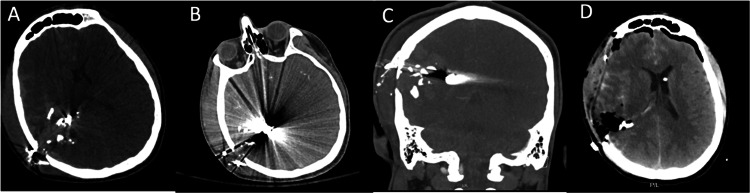
Preoperative axial and coronal CTH and postoperative axial CTH A) Axial CTH with a subdural window, B) axial CTH with the foreign body artifact, C) coronal CTH with a subdural window all demonstrating right parietal penetrating gunshot trajectory with associated subdural hematoma and multiple comminuted skull fragments with intraparenchymal hemorrhage in the parietal cortex with retained bullet fragment foreign bodies, D) postoperative axial CTH, showing improvement in the midline shift and superficial hematoma evacuation and left frontal interval EVD placement with the catheter tip terminating in the left lateral ventricle. CTH: computed tomography of the head

In March of 2023, a 31-year-old male presented to the emergency department with a reportedly self-inflicted right parietal penetrating gunshot wound with an associated subdural hematoma and a 7 mm midline shift (Figure 1).

The patient had a Glasgow Coma Scale (GCS) of 7 (E1VTM5), localizing with equal pupils in the emergency department (ED) on clinical examination with a Baylor bleeding score of 0. In addition, he was found to have an acidosis with a pH of 7.12 and hypothermia to a temperature of 28°C on arrival. Notably, the patient was covered in wet mud and had likely been down for six to eight hours prior to arrival at the ED. Initial vital signs were blood pressure (BP) 100-120/60-80, heart rate (HR) 60-70, relative risk (RR) 22, O_2_ saturation 100%, and temperature 29.2°C. CO_2_ was 47 on arrival and reduced to 41 on a repeat check after ventilatory adjustments. Labs included a creatinine kinase elevation at 372 units per liter, and white blood cell count was 26,000 per microliter (Table 1). 

**Table 1:** Patient laboratory values with normal range reference WBC: white blood count; INR: international normalized ratio; Hb: Hemoglobin

Laboratory values	Patient value	Normal range
pH	7.12	7.35-7.45
pCO_2_	47	35-45
Temperature	29.2°C	36.1-37.2°C
Creatinine kinase	372	24-204 U/L
WBC	26,000	4,500 to 11,000 WBC x10^9^/L
INR	1.1	0.8-1.1
Hb	14.3	14-18 g/dl

Thromboelestogram (TEG), platelets, and international normalized ratio (INR) were within normal limits. His initial hemoglobin was 14.3 g/dl. He was intubated, sedated, and taken urgently for right-sided decompressive craniectomy followed by external ventricular drain (EVD) placement for intracranial pressure monitoring. En route to the operating room, he received mannitol (1 g/kg); tranexamic acid 1 gm; triple antibiotic coverage including vancomycin, metronidazole, and cefepime [12]; levetiracetam for seizure prophylaxis; and propofol for sedation. The triple antibiotics were administered in accordance with the department protocol. Normal saline was also infused with pumps through two large-bore peripheral IVs. He was emergently transferred to the operating room for decompressive craniectomy and subsequent external ventricular drain placement. The initial ICP postoperatively was 9 mmHg.

Critical interventions in the ED included initiation of mannitol infusion, intubation, and mild hyperventilation to decrease intracranial pressure. A bolus of 1 g tranexamic acid was used to reduce bleeding and antibiotics (vancomycin, metronidazole, and cefepime) were provided to decrease the risk of infectious complications, levetiracetam for seizure prophylaxis, and propofol for sedation. On arrival in the operating room for right decompressive craniectomy, total intravenous anesthesia with propofol was administered to decrease cerebral metabolic rate and cerebral blood flow. Paralysis was provided to optimize surgical conditions and prevent shivering and narcotics for analgesia. The profound hypothermia was treated with a full-body forced-air warming blanket applied at the highest setting (46°C) and warming of all intravenous fluids. During the external ventricular drain placement, prior to the decompression craniectomy, additional mud and debris were removed from the patient’s body, the lines were organized, and a Foley catheter, arterial line, and a third peripheral IV were placed.

Anesthetic medications consisted of fentanyl 100 mcg, rocuronium 100 mg, propofol 100 mg (bolus), propofol 1170 mg (infusion), lasix 20 mg for suspected acute pulmonary edema, dexamethasone 8 mg, and calcium 1000 mg. During surgery, the patient's hemoglobin dropped to 7 gm/dl. Normal saline, Normosol, albumin 5%, and blood products were administered to maintain intravascular volume, normal vital signs, and adequate hemoglobin levels. Bicarbonate was deemed to not be indicated nor administered. Near the end of the case, arterial blood gas demonstrated improvement, but persistent severe acidosis to a pH of 7.20 and core temperature of 34°C. The patient remained intubated postoperatively and was transferred to the neurosurgical ICU for continued care and monitoring. Within three hours postoperatively, his pH normalized to 7.37 and the core temperature was brought to 37°C.

On postoperative day one, the patient regained consciousness and began following commands on the right side. His flap remained soft to palpation, and ICP measures were consistently under 22 mmHg postoperatively. A diagnostic cerebral angiogram was performed that was negative for intravascular injury. A first attempt was made at extubation on postoperative day 3, but the patient was shortly reintubated due to respiratory failure. A second attempt was made to extubate the patient on postoperative day 6 also resulting in subsequent reintubation for respiratory distress. It was decided that the patient would require a tracheostomy.

## Discussion

The present case demonstrates a young, otherwise healthy patient with TBI who required emergent operative intervention, decompressive craniectomy, and external ventricular drain placement, to prevent further brain injury and manage elevated intracranial pressures. His presentation was complicated by profound hypothermia and acidosis without discernable laboratory coagulopathy at presentation. Greater severity and duration of hypothermia are related to poorer outcomes [13-17]. Hypothermia has synergistic deleterious effects in combination with coagulopathy and acidosis that make up the lethal triad [13-17]. Early correction of hypothermia is paramount to avoid a potentially worsening vicious cycle of coagulopathy and metabolic disturbance. In addition, hypothermia reduces the release of oxygen from hemoglobin, which, in combination with increased peripheral vascular resistance, can contribute to tissue hypoxia [14]. Tissue hypoxia leads to anaerobic metabolism and lactate production and worsening acidosis [15]. Decreases in temperatures are also often observed during early resuscitation even in patients who are normothermic on arrival [1]. Some studies suggest that EDs may not have enough emphasis on correcting hypothermia, with only 30-60% of patients with core body temperature even recorded [3]. Consistent anatomical core temperature recording sites (tympanic membrane nasopharyngeal, esophageal, and bladder) [6] may also not be implemented, further disrupting the standard of care for hypothermia reversal.

Compensatory vasoconstriction and the sympathetic response work to reduce heat loss [18]. External application of warming devices generally is considered to prevent further heat loss while active rewarming requires invasive intervention measures [18]. As the patient warms, the peripheral vasculature dilates, lactate is released from peripheral tissues, the pH is subsequently lowered, and the liver and kidneys clear the acidosis over time [19]. More aggressive hyperthermia therapies, such as gastric, peritoneal, and bladder lavage with warm fluids, may cause a more rapid increase in lactate washout from cold and hypoperfused tissues than the liver is capable of metabolizing, leading to even more profound acidosis [4]. In addition, normothermia begins to optimize thermoregulatory thresholds and correct hypothermia-induced coagulopathy and thrombin formation [20].

In the setting of hypothermia, the compensatory mechanism of shivering causes oxygen demand to be significantly elevated, with an increase of 92% noted with a decrease in body temperature of only 1.2°C postoperatively [1]. This increase in cerebral metabolic rate of oxygen (CMRO_2_) can theoretically lead to further increases in intracranial pressure although it is uncertain whether cerebral blood flow is increased as a result. In addition, hypothermia inhibits thrombin clot initiation and fibrinogen synthesis [10]. Coagulopathy ensues at 33°C in in vitro studies, with synergistic coagulopathy from a mild acidosis at a pH of less than 7.26 [20]. Hypothermia can be profound, specifically when combined with general anesthesia [21,22]. During general anesthesia, vasodilation occurs, which causes a redistribution of heat from the core to the periphery, accounting for most heat loss [21,22]. The other causes of heat loss include radiation (60%), convection (30%), evaporation (<10%), and a negligible amount via conduction [22]. Proper management includes the prevention of further heat loss through the removal of wet clothing and the application of external warming blankets, heat padding, and elevated room temperature [15,23]. Active rewarming is achieved through warmed intravenous fluids or intra-cavitary lavage in severe hypothermia [4]. Avoidance of hypotension, electrolyte correction, and infection control are imperative, considering any other existing underlying conditions [24]. Other fluid solutions for resuscitation may have been considered, but normal saline has been shown to have reduced mortality compared with lactated ringer solution in TBIs [25]. In addition, while the routine use of tranexamic acid for TBI in general is not advised [26], it was used in the present study as a means to control bleeding in the setting of penetrating cranial trauma.

The primary goal of pH correction is to prevent myocardial dysfunction, vasopressor resistance, and arrhythmia [27]. Physiologic consequences of acidosis include increases in oxygen hemoglobin affinity, partial pressure carbon dioxide (PaCO_2_), and intracellular acidosis [27,28]. In cases where lactic acidosis is the underlying cause of acidemia, ionized calcium decreases (increased albumin binding) and anaerobic lactate production increases [28]. Thus, sodium bicarbonate administration worsens metabolic and respiratory acidosis. Even in cases of severe lactic acidosis (pH <7.20), the utility of sodium bicarbonate infusion to reduce morbidity and mortality has not been proven [29,30]. Given the patient’s severe acidosis, it was not administered.

Acute traumatic coagulopathy is identified in over 35% of cases of isolated TBIs [31].** **Hypofibrinogenemia is often altered in isolated TBIs and has been associated with higher inpatient mortality and serves as a prognostic outcome measure [32].** **In 2022, Sabouri et al. conducted a randomized controlled trial evaluating the effect of fibrinogen product administration in patients with severe TBI who had isolated hypofibrinogenemia [32]. The Glasgow Outcome Scale Extended was improved at 24 hours, 48 hours, and 72 hours with regression analysis indicating that hematoma and edema expansion correlated with a worse GCS score [32]. INR elevation from hypothermia has also been well described [4]. In the present case, coagulation studies, including INR and TEG (incorporating K-value and alpha angle, which detect hypofibrinogenemia), showed no abnormalities. Notably, tranexamic acid was given on arrival potentially altering the subsequent TEG measurement. Despite detectable alteration in coagulation studies, hypervigilance of potential coagulopathy (particularly with fibrinogen) is indicated in acute TBIs, especially in the setting of hematoma expansion, increases in edema, and neurological exam changes. Given the patient’s open, penetrating, unihemispheric ballistic injury with associated mass effect and midline shift (Baylor score of 0) that were thought to continue to worsen from penetrating injury and increased hematoma and edema, he was emergently taken to the operating room (OR) with concurrent administration of mannitol en route for ipsilateral decompressive craniectomy with contralateral external ventricular drain placement.

Practical considerations are also important to note as the patient was transported to the OR from the ED (Table 2).

**Table 2:** Practical management considerations for hypothermia in the traumatic, operative neurosurgery patient FAST: Focused Assessment with Sonography in Trauma; TEG: thromboelastogram; INR: international normalized ratio; PT: prothrombin time; PTT: partial thromboplastin time; ATLS: Advanced Trauma Life Support; abx: antibiotics; ECMO: extracorporeal membrane oxygenation

Heat loss prevention	Active rewarming	Laboratory	Miscellaneous
Removing wet clothes and mud from the body surface area Stopping active hemorrhage (i.e., FAST exam and tertiary survey). Consistent immediate recording of the core temperature on arrival.	Warmed IV fluid resuscitation, forced air warming, underbody warmer setup prior to pt arrival in the OR	Serial ABGs with appropriate correction of acidosis TEG, INR/PT/PTT, fibrinogen. Blood/wound samples sent for culture when clinically necessary.	Two large-bore IV lines (ATLS) consolidation of the abx regimen. Antibiogram to justify the use of abx, such as vancomycin. Perform wound dressing aseptically, a useful tool in the mitigation of the potentially existing infection. ECMO for severe, refractory hypothermia.

The removal of mud and alternate methods of rewarming could have been initiated sooner. A more timely and efficient approach to correcting hypothermia may have saved the patient from worsening acidosis. Alternatively, it may have overwhelmed the liver and kidneys and led to worsened lactic acidosis and cardiac arrest. In the ED, the removal of the wet mud coating the patient’s body and initiation of forced air warming could have been implemented immediately after measuring the core body temperature. Placing an underbody warmer on the operating table prior to patient transfer may have corrected core body temperature faster. Effective communication of vital signs between the ED and anesthesia providers improves preparation for OR setup. Once in the OR, forced air warmer, IV fluid warmer, and adequate coverage of the rest of the body parts not being operated on were successfully implemented.

After placement of two large-bore IVs following Advanced Trauma Life Support (ATLS) resuscitation protocol [33], additional IVs placed in the ED may be superfluous and could potentially slow OR start time. For example, the minimization of lines could be achieved through a consolidation of prophylactic antibiotics. The triple-antibiotic regimen was initiated on arrival, but a single coverage with cefazolin may be appropriately used for aerobic and anaerobic agents in the setting of penetrating ballistic injury [12,34]. In severe TBIs, mild induced hypothermia (35-36°C) has been considered to offer benefits in reducing the cerebral metabolic rate of oxygen while patients are prevented from shivering (via sedation/paralytic medications) [35]. Cerebral metabolism decreases by approximately 6-7% per degree Celsius [29]. Despite this, there is no definitive neuroprotective effect from hypothermia to incorporate into routine management in TBIs [36-38]. Severe hypothermia should be reversed as it is one of the most significant independent factors influencing both early and late mortality in ICU patients [3].

Future studies may investigate the timeframe that the TEG parameters may be altered due to hypothermia. Multimodal, aggressive, and potentially earlier rewarming may be investigated to ensure that further resuscitation efforts do not exacerbate hypothermia in the critically ill trauma patient.

## Conclusions

A case of severe hypothermia is described in a young patient with an operative ballistic penetrating cranial TBI with associated subdural hematoma. This case highlights the importance of early identification of hypothermia, coagulopathy, and acidemia with tips on how to effectively manage this lethal triad preoperatively, intraoperatively, and postoperatively. Notably, this case also highlights that coagulation studies (including the trauma TEG) may be within normal limits despite concurrent hypothermia and marked acidosis that propagate and exacerbate coagulopathy. Efficient and comprehensive management of hypothermia, acidosis, and coagulopathy mitigate perioperative risk in the setting of emergent operative intervention.
